# Community-based DOTS and family member DOTS for TB control in Nepal: costs and cost-effectiveness

**DOI:** 10.1186/1478-7547-6-20

**Published:** 2008-10-24

**Authors:** Tolib N Mirzoev, Sushil C Baral, Deepak K Karki, Andrew T Green, James N Newell

**Affiliations:** 1 Nuffield Centre for International Health and Development, Leeds Institute of Health Sciences, University of Leeds, Leeds LS2 9LJ, UK; 2 Health Research and Social Development Forum, PO Box 24133, Kathmandu, Nepal; 3 United Nations Population Fund (UNFPA), Nepal, UN House, Pulchowk, Lalitpur, PO Box 107, Kathmandu, Nepal

## Abstract

**Background:**

Two TB control strategies appropriate for South Asia (a community-based DOTS [CBD] strategy and a family-based DOTS [FBD] strategy) have been shown to be effective in Nepal in meeting the global target for the proportion of registered patients successfully treated. Here we estimate the costs and cost-effectiveness of the two strategies. This information is essential to allow meaningful comparisons between these and other strategies and will contribute to the small but growing body of knowledge on the costs and cost-effectiveness of different approaches to TB control.

**Methods:**

In 2001–2, costs relating to TB diagnosis and care were collected for each strategy. Structured and semi-structured questionnaires were used to collect costs from health facility records and a sample of 10 patients in each of 10 districts, 3 using CBD and 2 using FBD. The data collected included costs to the health care system and social costs (including opportunity costs) incurred by patients and their supervisors. The cost-effectiveness of each strategy was estimated.

**Results:**

Total recurrent costs per patient using the CBD and FBD strategies were US$76.2 and US$84.1 respectively. The social costs incurred by patients and their supervisors represent more than a third of total recurrent costs under each strategy (37% and 35% respectively). The CBD strategy was more cost-effective than the FBD strategy: recurrent costs per successful treatment were US$91.8 and US$102.2 respectively.

**Discussion:**

Although the CBD strategy was more cost-effective than the FBD strategy in the study context, the estimates of cost-effectiveness were sensitive to relatively small changes in underlying costs and treatment outcomes. Even using these relatively patient-friendly approaches to DOTS, social costs can represent a significant financial burden for TB patients.

## Background

Tuberculosis is a leading cause of death worldwide [1], South Asia being the worst affected region in terms of absolute numbers [2]. The internationally recommended DOTS strategy for TB control [3,4] has been successfully implemented in the public sector by many National Tuberculosis Programmes (NTPs) and there have been recent attempts to estimate the economic benefit of TB control at the international level [5]. A major element of the strategy is Direct Observation of Treatment (DOT), in which a health worker or someone responsible to the health system observes the patient swallowing each dose of treatment for at least the first 2 months of treatment (and preferably gives encouragement and support). In recent years there has been increasing awareness of the need to estimate the cost implications of various approaches to implementing DOTS [6-13]. This need is emphasised by the recent move to more flexible supervision and patient support within the new Stop TB strategy [14] and the International Standards for Tuberculosis Care (ISTC) [15].

In Nepal, over 14,000 new cases of smear positive (i.e. infectious) tuberculosis are notified each year [16]. Many patients in the so-called hill and mountain districts live far from the nearest health facility, so that access to daily DOT at a health facility is not feasible. To address this problem, two strategies – community-based DOTS and family-based DOTS – were developed and assessed in 10 districts using a district-randomised controlled trial (RCT), with each strategy being randomly assigned to 5 districts [17,18]. The only difference between the two strategies was the observation component, which comprises supervision by a community health volunteer or a family member respectively.

In the RCT, the treatment success rates of the CBD and FBD strategies were 85% and 89% respectively [18]. Both strategies have met the international target of 85% treatment success rate, and there was no significant difference between the outcomes of the two strategies. A detailed description of each strategy and the outcomes are published elsewhere [18]. The objective of this paper is to report the costs and cost-effectiveness of the two DOTS schemes in order to contribute to the future planning of TB control services in developing countries. It was important to assess not only health systems costs, but also those of patients, since with an average per capita income of US$378 [19], many patients in Nepal experience financial constraints to accessing TB treatment.

## Methods

Costing was undertaken in five districts of Nepal, namely Palpa, Syangja, Doti, Baglung and Dolakha. The first three implemented the CBD strategy and the remaining two the FBD strategy. The findings from Palpa district are discussed separately, as the district TB control programme in Palpa was supported by an NGO (United Mission to Nepal Tansen Hospital), which may have affected the study results.

The study used a comprehensive approach to estimating costs and cost-effectiveness, to allow a more holistic assessment of the long-term sustainability of the different strategies [20,21]. The data collected included not only costs to the health care system and direct costs to patients and their treatment supervisors but also social costs i.e. other costs (including opportunity costs) incurred by patients and supervisors. Analysis was initially planned to focus primarily on costs to the health system and include social costs for comparison purposes only. It was later decided to use sensitivity analysis on a model of total costs (including health systems and social costs) to better understand the study results.

The data for this study were collected in 2001–2002. All costing data was collected in Nepali Rupees (NRs) and then converted into US$ at the official exchange rate of the National Bank of Nepal for 2000–2001 of 1US$ = 74.65NRs.

Both start-up and recurrent costs were obtained. Start-up costs (primarily training and communication) were collected from health facilities using a structured questionnaire. Recurrent (ongoing) costs were collected across two main categories: costs to the health system and social costs. The costs to the health system were gathered from district health facilities' financial records using a structured proforma. Social costs (costs to the patients and their supervisors) were collected using semi-structured questionnaires. A sample of 50 patients (10 from each district) and their treatment supervisors was used. In each district, these 10 patients were selected randomly from patients under treatment during the period planned for data collection, using the district TB treatment register. Only patients who had completed the intensive phase of treatment (generally 2 months) and one month of the continuation phase of treatment were included, to ensure a wide coverage of costs.

In the analysis of recurrent costs, health system-related costs were allocated to six broad types, namely staff salaries, training (including monitoring and supervision), medicines, transportation, utilities and others (supplies, logistics, social mobilisation through DOTS committees), and to five levels: national, regional, district, treatment centre and treatment sub-centre. At each level only costs attributed to the organisation and management of the DOTS scheme were included. We also report start-up costs to allow an assessment of the feasibility of implementing such strategies.

Social costs were divided into those incurred directly by patients themselves and costs incurred by their treatment supervisors (community members in the CBD strategy and family members in the FBD strategy). Measures to minimise recall bias by patients and their supervisors included triangulation of data between patients and their treatment supervisors, triangulation methods applied during interviews, and informal discussion with local health care providers and community gate-keepers, to confirm details such as travel distances and number of visits to treatment centres. The social costs considered in this study included direct costs, opportunity costs and other (miscellaneous) expenses. Opportunity costs of time lost by patients and their supervisors were estimated as the actual time (in days) lost due to TB by patients and their supervisors for travel to health facility and for visits to the treatment supervisor/patient. The focus of this study was on costs of seeking treatment for TB and not on the economic impact of TB itself; therefore opportunity costs did not include the time lost due to patients being too ill to work.

A more detailed description of cost identification methods for each type of cost is given in Table 1.

**Table 1:** Methods of identification of costs in the study

**Costs**	**Notes**	**Identification method**	**Source of data**
*Health Systems costs*			

Staff salaries	Includes only the proportion of annual salary of staff attributable to TB	Salary rate for this category of staff (net salary per day) × annual days attributable to TB	Facility financial records Semi-structured questionnaire

Training	Includes monitoring and supervision	Cost of training for this intervention as a proportion of the total cost of training for each facility number of training courses in the study sites (for regional level)	Facility records Semi-structured questionnaire

Medicines		NTP estimates of medicine costs per patient × Total number of patients in the study sites	Tuberculosis Control in Nepal 2055–2060 (1998–2003), Long Term Plan; Annual Report of TB Control Program Nepal, 2058/2059 (2001/2002) Facility records (for No of patients)

Transportation	Includes transportation of medicines and laboratory supplies.	Total cost of transportation	Facility records

Utilities		Total cost of utilities × time for TB programme (e.g. 1/3 for regional level)	Facility records Semi-structured questionnaire

Others	Includes supplies, logistics, social mobilisation through DOTS committees		Facility records Semi-structured questionnaire

*Social costs*			

Direct costs	Includes treatment and travel charges	Number of visits × travel and consultation charges	Semi-structured questionnaire

Opportunity costs	Includes costs for time lost due to involvement in the scheme	Standardised daily rate for unskilled labour (NRs 85/day) × time lost due to involvement in the scheme	Semi-structured questionnaire

Other costs	Includes miscellaneous expenses such as refreshments while in the treatment centre	Patients and supervisors' recollection of any other expenses	Semi-structured questionnaire

Patients' and supervisors' costs were estimated per patient registered and then scaled up to the actual numbers of TB patients enrolled for treatment in each district according to NTP records (Table 2). Medians of costs were used when estimating social costs per patient as they are more representative of skewed data than means, especially when analysing small samples [9].

**Table 2:** Numbers of patients in each study district [18]

		Districts using community-based DOTS strategy			Districts using family-based DOTS strategy		
		
	Palpa	Syangja	Doti	Total*	Baglung	Dolakha	*Total*
Total no of patients treated, including:	422	335	125	*460*	136	117	*253*
No of patients successfully treated	367	276	106	*382*	104	104	*208*

* Total of Syangja and Doti

The effectiveness of each scheme was estimated using the number of patients for whom the treatment was successful [7]. To estimate cost-effectiveness, only recurrent costs were used, as we were primarily interested in assessing the long-term sustainability of the schemes although we recognise the limitations of excluding start-up costs, particularly in assessing the short-term cost implications of the scheme.

A sensitivity analysis of cost-effectiveness was performed to determine how the cost-effectiveness was sensitive to variations in the costs per patient and treatment success rates.

## Results

In the 5 districts used in the economic study, the total numbers of new sputum smear positive TB patients registered in the districts implementing the CBD and FBD strategies were 460 and 253 respectively. The total numbers of patients who were successfully treated were 382 and 208 respectively, giving treatment success rates of 83% and 82% respectively. A detailed breakdown of costs per patient is given in Table 3.

**Table 3:** Costs incurred in the districts using the community-based DOTS strategy and the family-based DOTS strategy (US$)

	Districts using the community-based DOTS strategy				Districts using the family-based DOTS strategy		
	
	Palpa	Syangja	Doti	Total*	Baglung	Dolakha	**Total**
Total number of patients treated	422	335	125	**460**	136	117	**253**
No of patients successfully treated	367	276	106	**382**	104	104	**208**
Treatment success rate	**87%**	82%	85%	**83%**	76%	89%	**82%**

							

**Costs per patient**							
Total recurrent costs, including:	**65.7**	71.4	89.3	**76.2**	85.4	82.5	**84.1**
Recurrent cost to health system	**42.0**	42.7	62.5	**48.1**	59.2	48.6	**54.3**
*Personnel costs*	*19.9*	*21.5*	*35.6*	*25.3*	*26.6*	*23.9*	*25.3*
*Drug costs*	*12.5*	*12.1*	*12.9*	*12.3*	*13.0*	*12.9*	*13.0*
*Transportation*	*0.9*	*1.1*	*6.8*	*2.7*	*5.1*	*3.2*	*4.2*
*Utilities*	*3.7*	*4.6*	*2.8*	*4.1*	*6.3*	*5.3*	*5.9*
*Training, supervision, monitoring*	*1.6*	*1.8*	*4.0*	*2.4*	*2.3*	*1.9*	*2.1*
*Others (supplies, logistics, social mobilisation)*	*3.5*	*1.6*	*0.5*	*1.3*	*5.9*	*1.4*	*3.8*
Recurrent cost to patients and supervisors	**23.6**	28.6	26.8	**28.1**	26.2	34.0	**29.8**
Total costs to patients:	**20.7**	25.2	24.4	**25.0**	21.6	19.2	**20.5**
*Opportunity costs*	*13.4*	*12.5*	*9.2*	*11.6*	*13.4*	*19.2*	*16.1*
*Direct costs*	*5.1*	*6.9*	*8.6*	*7.3*	*8.2*	*0.0*	*4.4*
*Other costs*	*2.2*	*5.8*	*6.6*	*6.1*	*0.0*	*0.0*	*0.0*
Total costs to supervisors, including:	**3.0**	3.4	2.3	**3.1**	4.6	14.7	**9.3**
*Opportunity costs*	*3.0*	*2.3*	*2.3*	*2.3*	*2.8*	*13.6*	*7.8*
*Direct costs*	*0.0*	*1.1*	*0.0*	*0.8*	*2.5*	*0.2*	*1.4*

* Total of Syangja and Doti

Total recurrent costs per patient were lower using CBD (US$76.2) than using FBD (US$84.1). In both CBD and FBD, social costs incurred by patients and their supervisors represented more than a third of total recurrent costs (37% in CBD and 35% in FBD).

Most health system-related costs were salaries (53% and 47% of total health system-related costs in CBD and FBD respectively) and medicines (26% and 24% respectively). A separate analysis of health system costs by programme level showed that about half the costs were incurred at the national level (US$22.3 and US$24.6 per patient in CBD and FBD respectively). The breakdown of health system-related costs by level (Table 4) shows the dominance of health systems-related costs at the national level (46% and 45% of total costs respectively) followed by district-level costs (32% and 16% respectively).

**Table 4:** Breakdown of health system-related costs by level (US$)

	Districts using the community-based DOTS strategy				Districts using the family-based DOTS strategy		
	
Level	Palpa	Syangja	Doti	Total*	Baglung	Dolakha	**Total**
Total, including	17,740.8	14,320.1	7,817.0	22,137.1	8,045.2	5,684.3	13,729.5
*National*	8,894.3	7,655.1	2,583.2	10,238.3	3,590.2	2,623.3	6,213.6
*Regional*	2,433.7	2,433.7	1,002.5	3,436.1	1,223.2	22.8	1,246.0
*District*	1,926.7	1,573.7	1,940.4	3,514.0	2,551.6	1,781.2	4,332.7
*Treatment centre*	3,904.7	1,324.8	1,149.7	2,474.5	431.1	903.0	1,334.1
*Treatment sub-centre*	581.4	1,332.8	1,141.3	2,474.2	249.0	354.0	603.0

* Total of Syangja and Doti

Patients' costs comprised the largest share of social costs (89% and 69% in CBD and FBD respectively). The majority of patients' costs were the opportunity costs of time lost due to seeking treatment for TB (46% and 79% respectively) followed by direct costs (29% and 21% respectively) and other costs (24% in CBD only). Likewise, most supervisors' costs were opportunity costs (73% and 84% respectively).

Start-up costs per patient registered were estimated as US$20 under the CBD strategy and US$31.7 under the FBD strategy.

The cost-effectiveness of the two strategies is given in Table 5. The total recurrent costs per case successfully treated were lower using CBD than using FBD. Health system-related costs dominated in both strategies (64% using CBD and 65% using FBD) with patient-related costs comprising most of the social costs (90% using CBD and 69% using FBD).

**Table 5:** Cost-effectiveness of the community-based DOTS strategy and the family-based DOTS strategy (US$)

	Community-based DOTS strategy				Family-based DOTS strategy		
	
	Palpa	Syangja	Doti	Total*	Baglung	Dolakha	**Total**
Recurrent costs per treatment succeeded, including	**75.5**	86.6	105.3	**91.8**	111.6	92.9	**102.2**
Total recurrent cost to health system per treatment succeeded	**48.3**	51.9	73.7	**58.0**	77.4	54.7	**66.0**
Total recurrent social costs (patients + supervisors) per treatment succeeded including:	**27.2**	34.7	31.6	**33.9**	34.3	38.2	**36.2**
*Total costs to patients per treatment succeeded*	*23.8*	*30.6*	*28.8*	*30.1*	*28.3*	*21.7*	*25.0*
*Total costs to supervisors per treatment succeeded*	*3.4*	*4.1*	*2.7*	*3.7*	*6.0*	*16.5*	*11.3*

* Total of Syangja and Doti

Unexpectedly, the costs and cost-effectiveness estimated for Palpa district were found to be lower than in the other two districts using the CBD strategy (Tables 3 and 5). This may be due to the different accounting systems and the subsidised structure of the TB treatment costs occurring in Palpa, a mission hospital.

**Figure 1:**
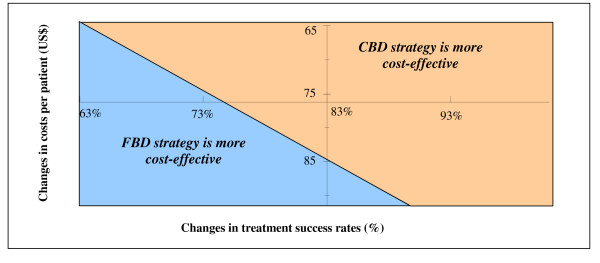
**Effect of changes in treatment success rates and costs per patient registered on the cost-effectiveness of the family-based DOTS and community-based DOTS strategies**. The figure presents the effect of changes in the treatment success rates (%) and costs per patient (US$) under the CBD strategy on the balance of cost-effectiveness between the FBD and CBD strategies. Note: The axes cross at the current treatment success rate (83%) and costs per patient (US$76.2) for the community-based DOTS strategy.

The effect of changes in costs per patient and treatment success rates in the CBD strategy on the balance of cost-effectiveness between CBD and FBD strategies is shown in Figure 1. Results of modelling of costs showed that a change of about 12% in either costs per patient treated or in the treatment success rate in one strategy can potentially shift the balance of cost-effectiveness between the CBD and FBD strategies.

## Discussion

The study results show that for both strategies, social costs make up more than a third of total costs. This fits with our expectation that social costs, particularly opportunity costs and direct (travel) costs are dependent on the distance between the patient and treatment supervisor, and is in line with findings from a study conducted in Bangladesh which found facility-based DOTS to be more expensive than a strategy involving Community Health Workers [22]. Thus, even though TB treatment is highly cost-effective, it can represent a considerable financial burden to patients, particularly economically disadvantaged groups. This finding is consistent with those from studies elsewhere [7,9,11].

We found that the CBD strategy was more cost-effective than the FBD strategy: recurrent costs per successful treatment were lower. This is in contrast to findings in Pakistan where family-based DOTS was found to be more cost-effective than a health centre/community-based scheme [8]. One explanation might be that CBD treatment success rates are higher. However, sensitivity analysis reveals that a change of 12% in either total costs per patient or a treatment success rate in either CBD or FBD strategy may shift the balance of cost-effectiveness between the strategies and make the FBD strategy more cost-effective.

Only recurrent costs have been considered in estimating cost-effectiveness, as the primary focus of this study is to assess the economic sustainability of the two approaches in the long term. We recognise, however, that start-up costs are also important, particularly to the health system. For example, the cost-effectiveness of developing similar initiatives elsewhere would depend on the availability of health staff at nearby health facilities (who will shoulder additional responsibilities associated with community-based initiatives).

Health systems costs per successful treatment under the CBD strategy were lower than under the FBD strategy. Similar results were found with start-up costs. These findings suggest that that FBD schemes are more expensive to establish and are likely to require larger recurrent resources from the health system than community-based schemes in the long-term. Start-up costs in the CBD strategy are effectively fixed costs and as such are unlikely to be very sensitive to the number of patients treated. On the contrary, start-up costs in the FBD scheme will generally be correlated with the number of patients.

An analysis of health systems costs by level showed a significant share of the costs was incurred at the national level. This indicates that budget absorption at the national level is high even in decentralised service delivery strategies such as CBD and FBD. We consider this an important finding, particularly when considering service delivery schemes at the local level as a means of reducing the financial burden to programmes at the national level.

Analysis of social costs per successful treatment reveals marginally lower costs for the CBD strategy than for the FBD strategy, supporting an earlier conclusion that it is more cost-effective to administer DOTS through community-based schemes. It is worth noting that these results are generally consistent with the difference in costs incurred by the health system. Within each arm, overall costs incurred by patients represent the largest share of overall social costs – an important finding if cost-minimisation strategies for economically disadvantaged populations are to be explored.

An unexpected finding is that the cost per successful treatment to family member treatment supervisors ($11.3) is three times that of community volunteer supervisors ($3.7). This is however mirrored by the finding that costs per successful treatment to patients on the FBD strategy ($25.0) are lower than those on the CBD strategy ($30.1) (Table 5). This almost certainly reflects a transfer of costs involved in travel to collect drugs, which in the family member strategy fall on the family member supervisor, and in the community strategy fall on the patient. It is important to note, however, that under the family member strategy, both patient and family member supervisor costs fall on the patient's family.

If the choice between CBD and FBD is guided by measures of cost-effectiveness, based on our study, we would recommend the CBD strategy. However, cost-effectiveness is by no means the only criterion for making any planning and policy decisions on TB control. Other criteria for such decisions may include the following:

• social acceptability of particular initiatives, e.g. willingness of patients to overcome possible cultural barriers in approaching a community member in order to request supervision of DOTS;

• availability of community member supervisors, as well as their attitude and willingness to monitor the treatment of TB patients;

• availability of robust supply and logistics mechanisms within the health system to ensure timely and continuous provision of DOTS medicines to patients/treatment supervisors;

• other health system- and social-related issues that are pertinent to different contexts such as the degree of decentralisation, or cultural norms which may facilitate or inhibit the implementation of decentralised DOTS schemes.

## Conclusion

The study found that the CBD strategy was more cost-effective than the FBD strategy in the context of the study districts of Nepal. However, cost-effectiveness was sensitive to relatively small changes in underlying costs and treatment outcomes. DOT at both community and family levels achieves high clinical outcomes (over 80%) at relatively low costs per successful treatment and both strategies or variants thereof are worthy of consideration by NTPs contemplating moving towards more patient-friendly supervision and patient support as recommended in the new Stop TB strategy [14] and the ISTC [15].

Social costs can represent a significant financial burden for TB patients, particularly if they are already poor. Cost-minimisation strategies should include consideration of social costs, to avoid situations where cost reductions merely shift costs from NTPs to patients. On the other hand, it is important to recognise the burden to the health system at the national level when planning decentralised strategies such as one assessed here.

Finally, despite the growing number of publications on the topic, further research using a consistent costing framework is needed to assess cost-effectiveness of similar forms of DOT (CBD and FBD) in other contexts as well as other forms of TB care (eg. self-administered treatment).

## Competing interests

The authors declare that they have no competing interests.

## Authors' contributions

TNM led the data analysis and coordinated the preparation of the manuscript; SCB participated in the design of the study, coordinated the data collection and participated in the data analysis and the preparation of the manuscript; DKK participated in the study design, led the data collection, participated in the analysis and preparation of the manuscript; ATG designed the study, advised on and participated in the data collection and analysis and participated in the preparation of the manuscript; JNN conceived of the study, participated in the design of the study, advised on and participated in the data collection and analysis and participated in the preparation of the manuscript. All authors have seen and approved the final version of the manuscript.
